# Gram Staining for the Treatment of Peritonsillar Abscess

**DOI:** 10.1155/2012/464973

**Published:** 2012-02-20

**Authors:** Yukinori Takenaka, Kazuya Takeda, Tadashi Yoshii, Michiko Hashimoto, Hidenori Inohara

**Affiliations:** ^1^Department of Otolaryngology-Head and Neck Surgery, School of Medicine, Osaka University Graduate, 2-1 Yamadaoka, Suita, Osaka 565-0871, Japan; ^2^Department of Otolaryngology, Kinki Central Hospital, 3-1 Kurumazuka, Hyogo, Itami 664-8533, Japan

## Abstract

*Objective*. To examine whether Gram staining can influence the choice of antibiotic for the treatment of peritonsillar abscess. *Methods*. Between 2005 and 2009, a total of 57 cases of peritonsillar abscess were analyzed with regard to cultured bacteria and Gram staining. *Results*. Only aerobes were cultured in 16% of cases, and only anaerobes were cultured in 51% of cases. Mixed growth of aerobes and anaerobes was observed in 21% of cases. The cultured bacteria were mainly aerobic *Streptococcus*, anaerobic Gram-positive cocci, and anaerobic Gram-negative rods. Phagocytosis of bacteria on Gram staining was observed in 9 cases. The bacteria cultured from these cases were aerobic *Streptococcus*, anaerobic Gram-positive cocci, and anaerobic Gram-negative rods. The sensitivity of Gram staining for the Gram-positive cocci and Gram-negative rods was 90% and 64%, respectively. The specificity of Gram staining for the Gram-positive cocci and Gram-negative rods was 62% and 76%, respectively. Most of the Gram-positive cocci were sensitive to penicillin, but some of anaerobic Gram-negative rods were resistant to penicillin. *Conclusion*. When Gram staining shows only Gram-positive cocci, penicillin is the treatment of choice. In other cases, antibiotics effective for the penicillin-resistant organisms should be used.

## 1. Introduction

Peritonsillar abscess is a localized accumulation of pus within the peritonsillar tissues, which usually results from acute tonsillitis and subsequent peritonsillar cellulitis. This disease is one of the most commonly encountered conditions in ear, nose, and throat (ENT) emergencies. It is characterized by sore throat, trismus, muffed voice, dehydration, dysphagia, and intense pain. Therefore, admission to the hospital is required for some patients with peritonsillar abscess. Intensive therapy may be required in some cases because it may lead to fatal complications, such as deep neck abscess and descending necrotizing mediastinitis [1].

 The treatment for peritonsillar abscess involves 2 steps: one is the removal of pus and the other is antibiotic therapy. For effective antibiotic therapy, we usually send the aspirates of the peritonsillar abscess for Gram staining and culture. However, previous reports have denied the effectiveness of bacteriologic studies [2–4].

 The aim of this study was to examine the efficacy of bacteriological studies of the peritonsillar abscess, with focus on the Gram-staining characteristics of the bacteria, and determine the value of this method in clinical practice.

## 2. Materials and Methods

A retrospective study was performed on peritonsillar abscess cases treated at Kinki Central Hospital between January 2005 and December 2009. There were 71 patients treated in that period. Of these patients, 62 received needle aspiration and 57 of the aspirates were sent for microbiological study. An 18-gauge needle was used to aspirate at the point of maximal fluctuation. Between 2005 and 2007, syringes were used for puncture and aspiration and immediately transported to the microbiology department. From 2008, the aspirates were injected into anaerobic container for transportation and storage. Then, the aspirates were processed for Gram staining, aerobic culture, and anaerobic culture. The results of culture studies were obtained for all 57 cases, while those of Gram staining were obtained for 45 cases. Low pathogenic oral flora were identified and reported only when they were predominant bacteria in the aspirates.

 The definition of phagocytosis on Gram staining is as follows: (1) the bacteria seen on the Gram staining are considered to be the same bacteria as the cultured bacteria, (2) more than several bacteria are in the phagocytes, and (3) the number of the bacteria in phagocytes is more than that around phagocytes.

 The sensitivity of cultured bacteria to ampicillin was determined by the Clinical and Laboratory Standards Institute disc method.

 Statistical analysis was performed using JMP software (SAS Institute Japan, Tokyo).

## 3. Results

**Figure 1:**
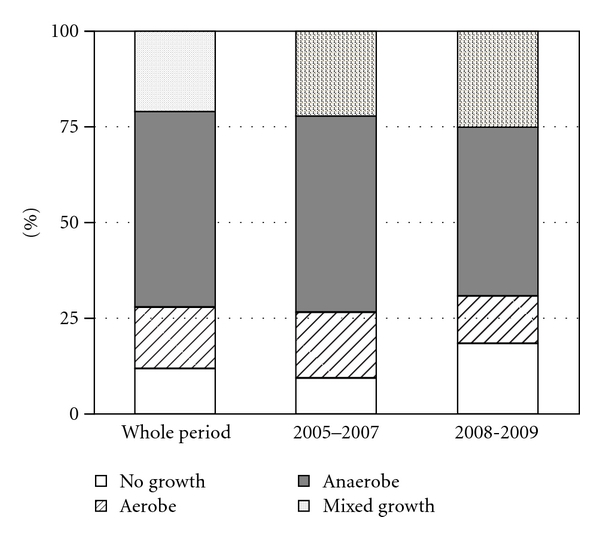
Results of bacteriological study for whole period, period between 2005 and 2007, and period between 2008 and 2009. Results of bacterial culture were classified as no growth, aerobes only, anaerobes only, and mixed growth of aerobes and anaerobes.

Results of the 57 culture studies were demonstrated in Figure 1. During the whole period, no bacteria was cultured in 12% of the cases, only aerobes in 16%, only anaerobes in 51%, and both aerobes and anaerobes in 21%. Namely, anaerobes were cultured in 72% of cases. Next, we examined the influence of transportation and storage method. The culture results between 2005 and 2007, during which syringes were used as container, and those between 2008 and 2009, during which anaerobic container were used as container, were also shown in Figure 1. The percentages of anaerobe cultured cases in early period were comparable with those of later period (73% and 69%, resp.), and there was no statistically significant difference. This implies that use of anaerobic container has little effect on the result of culture studies.

 Further, 83 pathogens were identified in the 50 cases (Table 1). Of the isolated aerobes, 76% (19 out of 25 aerobes) were Gram-positive cocci (GPC). The most common aerobe was *Streptococcus pyogenes*. It was isolated from 20% of cases. In contrast to aerobes, of which GPC were predominant, GPC and Gram-negative rods (GNRs) were frequently observed among anaerobic pathogens. The most frequent Gram-positive anaerobe was anaerobic *Streptococcus* (30% of cases). The common anaerobic Gram-negative rods were *Fusobacterium *(26%), *Prevotella* (22%), *Bacteroides* (8%), and *Porphyromonas *(8%). It is noteworthy that the frequency of *Staphylococcus aureus*, *Streptococcus pneumoniae*, and *Haemophilus influenzae* was similar to those of low pathogenic oral flora (i.e., *Neisseria*, *Lactobacillus*, and *Bifidobacterium*).

 To identify the pathogen that causes peritonsillar abscess, we examined the phagocytosis of bacteria by Gram staining. Phagocytosis of bacteria is observed if the bacteria is a pathogen [5]. Phagocytosis was observed in 9 cases out of 45 cases, in which both Gram staining and culture study were performed. The aspirates were obtained before the administration of antibiotics in 3 out of the 9 cases, while the aspirates were obtained after the initiation of antibiotic therapy in the other cases. The cultured organisms from phagocytosis-positive cases are shown in Table 2. Only aerobic streptococci, anaerobic GPC, and anaerobic GNR were detected. From the results of Tables 1 and 2, we assume that aerobic and anaerobic streptococci and GNR (*Prevotella*, *Fusobacterium*, *Bacteroides*, and *Porphyromonas*) are the main causative pathogens of peritonsillar abscess.

 To determine whether Gram staining is useful to identify the pathogens, we compared the result of Gram staining with that of cultured isolates. Among the 45 cases, of which the results of both culture study and Gram staining were obtained, GPC, GPR, GNC, and GNR were cultured in 21 cases, 3 cases, 1 case, and 28 cases, respectively. We used the result of bacterial culture as the reference standard and calculated the sensitivity, specificity, positive predictive value, and negative predictive value of Gram staining (Table 3). In Table 3, the results of GPC and GNR, as causative pathogens, were important. The sensitivity and specificity of Gram staining to detect GPC were 90% (19/21) and 62% (15/24), respectively; further, its sensitivity and specificity to detect GNR were 64% (18/28) and 76% (13/17), respectively. These results suggest that Gram staining is fairly reliable method. Furthermore, positive predictive value of Gram staining for GNR is as high as 82%, demonstrating that antibiotic therapy against GNR is indispensable when GNR is detected on Gram staining.

 Next, we examined whether penicillin can be the treatment of choice for peritonsillar abscess. The sensitivity of the main pathogens to ampicillin is shown in Table 4. Most aerobic and anaerobic GPC were sensitive to penicillin. In contrast, anaerobic GNRs were moderately resistant to penicillin. This result demonstrates that penicillin is effective only when GNR is not present.

## 4. Discussion

Previous culture studies of peritonsillar abscess aspirates show wide variety of pathogens, including both aerobes and anaerobes. Anaerobes were isolated from 72% of examined aspirates in this study. However, the proportion of anaerobes varies widely among studies (33%–83%) [3, 6–9]. One reason for the variation is the difference in microbiological handling and culture techniques used. Anaerobic bacteria are easily killed by brief exposure to air during sampling, transport, or processing. Therefore, the aspirates should be transported under anaerobic condition and immediately streaked on plates for culture. However, immediate processing is sometimes difficult, especially out of normal laboratory hours. We started injecting the aspirates into anaerobic containers for transportation and storage from 2008. Before 2008, we were using syringes, for puncture and aspiration, as containers. Therefore, we might have missed certain proportion of anaerobes before 2008, although the storage and handling method made no apparent difference (Figure 1). The other reason for the variation among bacteriological studies is that handling of oral bacterial flora differs among different laboratories. The aspirates usually contain pathogens as well as oral flora. Some laboratories neither isolate nor report the oral bacterial flora, assuming that these bacteria are not pathogens of peritonsillar abscess. Other laboratories isolate, identify, and report the oral bacterial flora, assuming that these bacteria are possible pathogens. However, we did not have an exact idea of which bacteria should be cultured, isolated, and identified, because not only highly pathogenic bacteria, like *Streptococcus pyogenes*, but also oral flora bacteria may cause peritonsillar abscess. We did not know whether *H. influenzae* could be the pathogen of peritonsillar abscess. 

 Phagocytosis is a major mechanism used to remove pathogens. Phagocytes eliminate the pathogens by engulfing it. The phagocytosed bacteria can be identified as intracellular bacteria within phagocytes on Gram staining. When specimens for bacterial studies were obtained from the place where potential pathogens are colonizing, culture or microscopic examination is of limited value because pathogens as well as colonized bacteria are detected. In such circumstances, Gram staining can be of diagnostic value since phagocytosed bacteria on Gram staining are regarded as pathogens [5]. By examining phagocytosis on Gram staining and comparing with the culture results, we elucidated here that *S.pyogenes*, other streptococci, and anaerobic GNR (*Prevotella*, *Bacteroides*, *Fusobacterium*, and *Porphyromonas*) are causative pathogens of peritonsillar abscess. The problem here is that the percentage of phagocytosis positive cases was as low as 20% (9 cases out of 45 cases). One of the reason may be that the criteria for the definition of phagocytosed bacteria was too strict in this study. There is no common definition of the phagocytosis on Gram staining, and, in most reports, qualitative description is used for its definition [10–12]. The other reason is that a substantial proportion of patients (21 out of 57 patients) had received antibiotic therapy before needle aspiration. Antibiotic treatment has significant effect on the result of Gram staining [13].

 Although more than half of ENT doctors recommend microbiological examination of peritonsillar abscess aspirates, a large number of them are not routinely followed up [2]. Cherukuri and Benninger indicated that bacteriologic studies are unnecessary on initial presentation in the routine management of peritonsillar abscess [4]. Repanos et al. reported that the treatment course was not changed for any patient in their study based on the results of microbiological studies [3]. The main reason is that the growth of anaerobes is so slow that the culture reports are not usually available during the hospitalization period. Therefore, we decided to study their Gram-staining characteristics. Although Gram staining divides the bacteria to only 4 groups, it is an easy procedure and is not time consuming. The results can be obtained within an hour. Furthermore, Gram-staining reliably predicts the types of bacteria (Table 3).

 Penicillins were the first choice of antibiotics in treating peritonsillar abscess [9, 14, 15]. Snow et al. showed that penicillin is effective in the majority of cases and that it should be used as the initial antibacterial agent in nonallergic patients [14]. Ophir et al. assumed that removal of pus containing high levels of beta-lactamase enables the subsequently administered penicillin to eradicate the susceptible bacteria, thus accounting for the remarkably high success rate of using penicillin in their study in 1998 [16]. However, the situation is different today. The increase of beta-lactamase-producing organisms has limited the use of penicillin. In view of the mixed flora that cause peritonsillar abscess and the increasing number of beta-lactamase-producing microorganisms, the use of antibiotics active against beta-lactamases has become more popular in clinical practice [17]. For these reasons, the antibiotics recommended today are clindamycin, augmented penicillin, and either penicillin or cephalosporin plus metronidazole [18, 19]. However, if we distinguish penicillin-sensitive organisms from penicillin-resistant organisms at the time of initial treatment, penicillin can still be a good choice. The key is the Gram-staining properties of the bacteria. Megalamani et al. reported that the presence of gram-negative bacteria warrants the use of antibiotics other than penicillin [20]. Many of the anaerobic GNR are beta-lactamase producers, especially *Bacteroides*, *Prevotella*, and some *Fusobacterium* species [21, 22]. Penicillin has poor activity against *Bacteroides* sp. and *Prevotella*. It has only moderate activity against *Fusobacterium* and exceptionally good activity against *Porphyromonas*. Therefore, the anaerobic GNR are resistant to penicillin in general. However, both streptococci and anaerobic GPC show high susceptibility to penicillin [21, 22]. The susceptibility of GPC to penicillin was also confirmed in our study (Table 4). Therefore, we have to check whether the aspirates contain GNR (penicillin-resistant organisms). Gram staining is the easy and rapid method to check it. Thus, we propose the strategy depicted in Table 5. We send the aspirates to microbiology laboratory and then check the result of Gram stain. If Gram staining shows only GPC, penicillin is the choice of treatment. Otherwise, clindamycin, augmented penicillin, and either penicillin or cephalosporin plus metronidazole should be used.

## 5. Conclusion

GPC (both aerobic and anaerobic) and anaerobic GNR are causative pathogens of peritonsillar abscess. These GPC were sensitive to penicillin, while some of GNR were resistant to penicillin. Therefore, Gram staining can determine the choice of antibiotics.

## Figures and Tables

**Table 1:** Isolated organisms from the aspirates.

	No. of isolates	% of cases		No. of isolates	% of cases

Aerobes			Anaerobes		

Gram-positive cocci			Gram-positive cocci		
*Staphylococcus aureus *	2	4	* Anaerobic Streptococcus*	15	30
* Streptococcus pneumoniae*	1	2	* Peptococcus*	1	2
*Streptococcus pyogenes *	10	20			
**α*-Streptococcus *	5	10			
**β*-Streptococcus *	1	2			

Gram-positive rods			Gram-positive rods		
*Corynebacterium sp. *	1	2	* Bifidobacterium *	1	2
			* Lactobacillus*	1	2

Gram-negative cocci			Gram-negative cocci		
*Neisseria *	2	4	* Veillonella*	1	2

Gram negative rods			Gram-negative rods		
*Haemophilus influenzae *	2	4	Bacteroides*	7	12
*Haemophilus parainfluenzae *	1	2	* Fusobacterium*	13	26
			Prevotella*	12	22
			* Porphyromonas*	4	8
			* Capnocytophaga *	1	2

Others					
* Candida albicans*	2	4			

*Two strains were cultured in one case.

**Table 2:** Cultured organisms from phagocytosis—positive cases.

	No. of cases
Aerobic Gram-positive cocci	
*Streptococcus pyogenes *	3
*α*-*Streptococcus *	1

Anaerobic Gram-positive cocci	
* Anaerobic Streptococcus*	4
* Peptococcus*	1

Anaerobic Gram-negative rods	
* Prevotella*	2
* Fusobacterium*	1
* Bacteroides*	1
* Porphyromonas*	1

**Table 3:** Clinical usefulness of Gram staining.

	Gram-positive cocci	Gram-positive rods	Gram-negative cocci	Gram-negative rods

Sensitivity (%)	90	100	0	64
Specificity (%)	62	69	100	76
Positive predictive value (%)	68	19	0	82
Negative predictive value (%)	88	100	98	57

**Table 4:** Sensitivity of pathogens to penicillin.

	% of ampicillin sensitive strains	

Gram-positive cocci		
*Steptococcus pyogenes *	100	(10/10)

Anaerobic Gram-positive cocci		
* Anaerobic Streptococcus*	92.3	(12/13)
* Peptococcus*	100	(1/1)

Anaerobic Gram-negative rods		
* Bacteroides *	71.4	(5/7)
* Fusobacterium*	83.3	(10/12)
* Prevotella*	100	(11/11)
* Porphyromonas*	75	(3/4)

**Table 5:** Gram staining and recommended antibiotics.

Gramstaining	Expected pathogen	Recommended antibiotics
GPC	Aerobic, anaerobic streptococci	penicillin

Negative GNR GPC + GNR	Aerobic, anaerobic streptococci Anaerobic GNR (*Prevotella*, *Fusobacterium*, *Bacteroides*, *Porphyromonas*)	Clindamycin Penicillin Cephalosporin plus metronidazole
